# Association of Maternal Bone Resorption with Urinary Fluoride Levels During Pregnancy

**DOI:** 10.3390/toxics14090798

**Published:** 2026-09-09

**Authors:** Morteza Bashash, Karen E. Peterson, E. Angeles Martinez-Mier, Héctor Lamadrid-Figueroa, Ángel Santiago, Adrienne S. Ettinger, Martha María Téllez-Rojo, Howard Hu

**Affiliations:** 1 Department of Population and Public Health Sciences, Keck School of Medicine, University of Southern California, Los Angeles, CA 90033, USA; 2 Department of Nutritional Sciences, University of Michigan School of Public Health, Ann Arbor, MI 48109, USA; 3 Department of Dental Public Health and Dental Informatics, Indiana University School of Dentistry, Indianapolis, IN 46202, USA; 4 Department of Perinatal Health, Instituto Nacional de Salud Pública, Cuernavaca 62100, Morelos, Mexico; 5 Rutgers Health, Rutgers University, Newark, NJ 07107, USA; 6 Center for Nutrition and Health Research, Instituto Nacional de Salud Pública, Cuernavaca 62100, Morelos, Mexico

**Keywords:** fluoride, maternal urinary fluoride, bone resorption, N-telopeptide (NTX), pregnancy, prenatal exposure, bone turnover, ELEMENT cohort, neurodevelopmental toxicant

## Abstract

**Highlights:**

**What are the main findings?**

- Maternal bone resorption was independently and positively associated with maternal urinary fluoride in each trimester of pregnancy. This is consistent with fluoride stored in the maternal skeleton being released into the body as bone breaks down during pregnancy.

**What is the implication of the main finding?**

- Maternal urinary fluoride during pregnancy appears to reflect both external fluoride exposure and endogenous fluoride released from the maternal skeleton during bone resorption. This has implications for how prenatal fluoride exposure is measured and interpreted, since urinary fluoride may capture a bone-derived component in addition to recent exposure.

**Abstract:**

Evidence is accumulating linking prenatal fluoride exposure with adverse neurodevelopment. Maternal urinary fluoride (MUF) is a well-established biomarker of fluoride exposure. Because most fluoride is stored in calcified tissues, pregnancy-associated changes in bone metabolism may contribute to fetal fluoride exposure. We evaluated whether maternal bone resorption, indexed by urinary N-telopeptide of type I collagen (NTX), is associated with MUF across pregnancy. Participants were drawn from the Early Life Exposures in Mexico to Environmental Toxicants (ELEMENT) birth cohorts. Each woman contributed one paired MUF–NTX measurement per trimester. Both biomarkers were creatinine-normalized to account for urine dilution. Trimester-specific generalized linear models with a Gamma distribution estimated associations between NTX and MUF, adjusting for maternal age, birth order, infant birthweight, and cohort. Across the trimesters, MUF was relatively stable (0.89 ± 0.37, 0.91 ± 0.41, and 0.83 ± 0.42 mg/L), whereas NTX increased with gestation (68.0 ± 33.5, 99.0 ± 50.3, and 132.4 ± 65.4 nmol BCE/mM creatinine). Higher NTX was consistently associated with higher MUF: β = 0.00315 (*p* < 0.0001) in Trimester 1, β = 0.00207 (*p* = 0.001) in Trimester 2, and β = 0.00253 (*p* < 0.0001) in Trimester 3, corresponding to 11%, 11%, and 18% higher MUF per 1-SD increase in NTX. Results were unchanged after adjustment for estimated fluoride intake, with no evidence of non-linearity. Maternal bone resorption was independently associated with MUF, consistent with a skeletal contribution to urinary fluoride. Research may be needed on measures that can blunt bone mobilization of fluoride during pregnancy.

## 1. Introduction

Fluoride exposure occurs through drinking water, salt, dental products, and a variety of foods, and a growing body of evidence now identifies fluoride as a potential developmental neurotoxicant. Prospective cohort studies have reported associations between higher prenatal fluoride exposure and adverse neurobehavioral outcomes in children, including lower cognitive scores and increased symptoms of inattention and hyperactivity [1,2,3,4,5,6]. Recent reviews and the National Toxicology Program’s monograph highlight consistent signals linking prenatal fluoride exposure with neurodevelopmental risk [7,8,9].

Interpreting maternal urinary fluoride (MUF) as a biomarker of fetal fluoride exposure is complicated by the fact that fluoride originates from both external intake and endogenous release from bone [10,11]. Fluoride accumulates extensively in calcified tissues, with approximately 95–99% of total body fluoride stored in the skeleton [12,13]. Because fluoride incorporates into hydroxyapatite during bone formation, the maternal skeleton functions as the primary internal reservoir of fluoride throughout life [14]. When bone undergoes resorption, fluoride embedded in the mineral matrix is released into circulation and ultimately excreted in urine [15].

Pregnancy is characterized by dynamic changes in maternal calcium and bone metabolism [16]. To support fetal skeletal development, maternal physiology enhances intestinal calcium absorption and, when insufficient, increases bone resorption, particularly in mid- and late gestation [17,18,19,20]. These physiologic increases in osteoclastic activity create a plausible mechanism through which endogenous fluoride could be released from maternal bone into the bloodstream at heightened rates during pregnancy [10]. This same mechanism has been noted to be responsible for the pregnancy-related increases in blood levels that have been observed with respect to lead [21,22], another element known to accumulate in the hydroxyapatite of bone.

N-telopeptide of type I collagen (NTX) is a well-established biochemical marker of bone metabolism that is specific for resorption and increases when osteoclastic activity accelerates [23,24]. Because NTX reflects the intensity of bone breakdown, it offers a biologically relevant indicator of potential endogenous fluoride release. If fluoride stored in bone is mobilized during pregnancy-related bone resorption, then MUF should correlate with NTX within each trimester. The relationship between bone turnover and urinary fluoride excretion has received limited empirical investigation, despite its relevance for interpreting fluoride biomarkers in pregnancy and for understanding endogenous fluoride contributions to fetal exposure.

Given the recognition of fluoride as a potential neurodevelopmental toxicant and the biological mechanism linking bone resorption to internal fluoride release, we evaluated whether maternal NTX is associated with MUF during pregnancy. Using trimester-specific datasets, we tested the hypothesis that higher NTX corresponds to higher MUF, consistent with physiologic mobilization of fluoride from maternal bone during periods of increased skeletal turnover.

The overall objective of this study was to determine whether endogenous skeletal fluoride, mobilized through bone resorption during pregnancy, contributes to maternal urinary fluoride independently of external fluoride intake. Our specific aim was to quantify the trimester-specific association between maternal bone-resorption activity, indexed by urinary NTX, and creatinine-adjusted MUF. We tested the primary hypothesis that, within each trimester, higher NTX is associated with higher MUF, consistent with the release of skeletal fluoride during periods of increased bone turnover, and the secondary hypothesis that this association persists after adjustment for estimated dietary fluoride intake. Confirming these hypotheses would establish that MUF reflects both recently ingested and endogenously released fluoride, with direct implications for the interpretation of maternal fluoride biomarkers in studies of prenatal exposure and child neurodevelopment.

## 2. Materials and Methods

Participants were drawn from the Early Life Exposures in Mexico to Environmental Toxicants (ELEMENT) study, a longitudinal pregnancy cohort program in Mexico City [1,2]. Cohort 2 represents an observational cohort enrolled during early gestation, and Cohort 3 consists of participants from a randomized trial of calcium supplementation, assigned to either placebo (Cohort 3 Ca–) or calcium supplementation (Cohort 3 Ca+). All cohorts followed standardized procedures for recruitment, urine collection, and maternal interviews. Each participant was solicited to contribute one paired MUF–NTX measurement per trimester. Trimester-specific models used only complete-case observations, and sample sizes differed by trimester accordingly.

The ELEMENT project comprises a series of successively enrolled longitudinal birth cohorts of mother–child pairs recruited from maternity hospitals in Mexico City that serve low- to moderate-income populations [1,25]. Eligible participants were healthy women with a normal singleton pregnancy who provided written informed consent prior to enrollment, and all cohorts followed standardized protocols for recruitment, urine collection, and maternal interview [1,25]. The present analysis used the two cohorts with archived maternal pregnancy urine and repeated trimester-specific biomarker measurements: Cohort 2, an observational pregnancy cohort originally established to examine prenatal lead exposure and child neurodevelopment, and Cohort 3, a double-blind randomized trial in which women were assigned during the first trimester to 1200 mg/day calcium (Cohort 3 Ca+) or placebo (Cohort 3 Ca–) [22,26].

Maternal urinary fluoride (MUF), measured in mg/L and creatinine-adjusted using the trimester-specific protocol previously applied in the ELEMENT cohorts [1], served as the outcome. MUF was quantified from a single second-morning-void urine specimen collected at each trimester [1]. Urinary N-telopeptide of type I collagen (NTX), reported in nmol BCE per millimolar of creatinine (nmol BCE/mM creatinine)—the laboratory-standard creatinine-normalized scale on which the assay is delivered and the convention in the bone-turnover literature—served as the exposure and was quantified from the same urine sample using a validated immunoassay [21]. Because MUF and NTX were measured in the same second-morning-void specimen, raw concentrations of both analytes are jointly influenced by hydration, which can induce a spurious positive correlation in the absence of a biological link; creatinine-adjusting both biomarkers prior to modeling forecloses urine dilution as a shared cause of any observed MUF–NTX association. NTX values were trimmed using a distribution-based rule, whereby observations exceeding 3.5 standard deviations from the trimester-specific mean were excluded prior to analysis.

Maternal urinary fluoride was measured in the same archived second-morning-void specimens using a modified hexamethyldisiloxane (HMDS)-facilitated microdiffusion method coupled with an ion-selective electrode, at the University of Michigan School of Public Health and the Indiana University Oral Health Research Institute [1]. In brief, 3 M sulfuric acid saturated with HMDS was added to each sample, and fluoride was allowed to diffuse for 20–24 h and was trapped in sodium hydroxide; the trapped fluoride was then neutralized with acetic acid and quantified using a fluoride ion-selective electrode and pH/ISE meter (Thermo Scientific Orion; Thermo Fisher Scientific, Waltham, MA, USA), with concentrations derived from a standard curve and all analyses performed in a clean room. Quality-control procedures included daily instrument calibration, procedural blanks, replicate runs, and certified reference materials (Institut National de Santé Publique du Québec; NIST 3183 Fluoride Anion Standard [27]). Fluoride concentrations were divided by urinary creatinine to account for urine dilution [1].

Urinary NTX was measured in the same second-morning-void specimen used for MUF, collected at each trimester visit. NTX is a specific marker of osteoclast activity (bone resorption) that has been shown to be stable and resistant to degradation in stored samples. Samples were analyzed with a commercially available competitive-inhibition enzyme-linked immunosorbent assay (Osteomark; Ostex International, Seattle, WA, USA). NTX concentrations were controlled for urine dilution using creatinine concentration and expressed as nanomoles of bone collagen equivalents (BCE) per millimole of creatinine (nmol BCE/mM creatinine). The intra-assay coefficient of variation was 8.9% (at 406 nmol BCE) and 8.7% (at 1563 nmol BCE), and the inter-assay coefficient of variation was 8.6% (at 427 nmol BCE) and 5.6% (at 1513 nmol BCE) [22,26].

Covariates selected a priori included maternal age (continuous), firstborn status (binary), cohort membership (Cohort 3 Ca– as the reference, Cohort 3 Ca+, Cohort 2), and infant birthweight (delivery weight, continuous). Covariates were chosen based on biological relevance and availability across trimesters.

Descriptive statistics were calculated for all variables. MUF showed right-skewness and non-constant variance; therefore, MUF was modeled using Generalized Linear Models with a Gamma distribution and log link, with robust standard errors. Models were estimated for each trimester with MUF as the dependent variable and trimester-specific NTX as the primary predictor. The effect of a 1-standard deviation increase in NTX was calculated, and differences in MUF (mg/L) were derived from trimester-specific MUF means. Model diagnostics included inspection of residual distributions, fitted–residual patterns, and influence statistics. Generalized additive models (GAMs) were used to visualize the adjusted association between NTX and MUF and to assess potential non-linearity in the exposure–outcome relationship. GAM plots were compared with the linear predictor from the primary Gamma model to determine whether a non-linear pattern was present. Each model was fit within a single trimester. Non-linearity was tested by a quadratic NTX term; sensitivity analyses excluded the calcium-supplemented cohort, removed birthweight or replaced it with early-pregnancy maternal BMI, and compared included versus excluded participants (Tables S1–S3). All tests were two-sided with a significance level of *p* < 0.05.

All analyses were performed in Stata 19 (StataCorp LLC, College Station, TX, USA) and R version 4.5.1 (R Foundation for Statistical Computing, Vienna, Austria).

Fluoride intake was estimated from water, beverages, and commonly consumed foods to account for external sources of exposure in Mexico, where salt fluoridation and variable fluoride concentrations in commercial drinks contribute to overall intake. Intake estimates were derived from reported consumption patterns and the fluoride content of drinking water and frequently consumed beverages and foods [28,29]. These values were summed to generate an approximate total daily fluoride intake for each participant. This variable was included in sensitivity analyses to evaluate whether external fluoride exposure altered the association between NTX and MUF.

## 3. Results

Crude MUF measurements were available for 659, 529, and 381 participants in trimesters 1, 2, and 3, and urinary NTX in 699, 708, and 686 participants, of whom 444, 203, and 250 also had a creatinine-adjusted MUF. Trimming removed values exceeding the trimester-specific 3.5-SD threshold, and complete-case restriction to all model covariates yielded trimester-specific analytic sample sizes of 423 for the first trimester, 197 for the second trimester, and 239 for the third trimester. This retained ≥95% of participants with a creatinine-adjusted MUF, and included and excluded participants did not differ in age, birthweight, or firstborn status (Table S1). Mean maternal age was approximately 26.5 years across trimesters, and mean infant birthweight was about 3.1 kg. About one third of children were firstborn (35–38% across trimesters). In the combined longitudinal analytic sample (*n* = 506), 36.6% of participants were in Cohort 3 Ca–, 34.6% in Cohort 3 Ca+, and 28.9% in Cohort 2. Descriptive characteristics by trimester and for the full analytic sample are shown in Table 1.

MUF concentrations were relatively stable between the first and second trimesters and slightly lower in the third trimester. Mean (SD) MUF was 0.89 (0.37) mg/L in Trimester 1, 0.91 (0.41) mg/L in Trimester 2, and 0.83 (0.42) mg/L in Trimester 3. In contrast, NTX increased over the course of pregnancy. Mean (SD) NTX was 68.01 (33.54) nmol BCE/mM creatinine in Trimester 1, 99.01 (50.32) nmol BCE/mM creatinine in Trimester 2, and 132.36 (65.41) nmol BCE/mM creatinine in Trimester 3. The combined longitudinal analytic sample had a mean (SD) MUF of 0.89 (0.38) mg/L and a mean (SD) NTX of 69.93 (35.75) nmol BCE/mM creatinine (Table 1).

In Gamma regression models, higher NTX was consistently associated with higher MUF within each trimester (Table 2). In Trimester 1, NTX showed a strong positive association with MUF (β = 0.00315; *p* < 0.0001). A 1-SD increase in NTX (33.54 nmol BCE/mM creatinine) corresponded to an 11% higher MUF concentration (ratio = 1.11), which is equivalent to an absolute increase of approximately 0.10 mg/L relative to the Trimester 1 mean. In Trimester 2, the association remained strong (β = 0.00207; *p* = 0.001). A 1-SD increase in NTX (50.32 nmol BCE/mM creatinine) was associated with an 11% higher MUF (ratio = 1.11), corresponding to an absolute increase of about 0.10 mg/L relative to the Trimester 2 mean. In Trimester 3, the association was the largest of the three trimesters and remained statistically significant (β = 0.00253; *p* < 0.0001). A 1-SD increase in NTX (65.41 nmol BCE/mM creatinine) was associated with an 18% higher MUF (ratio = 1.18), corresponding to an absolute increase of about 0.15 mg/L relative to the Trimester 3 mean.

**Figure 1:**
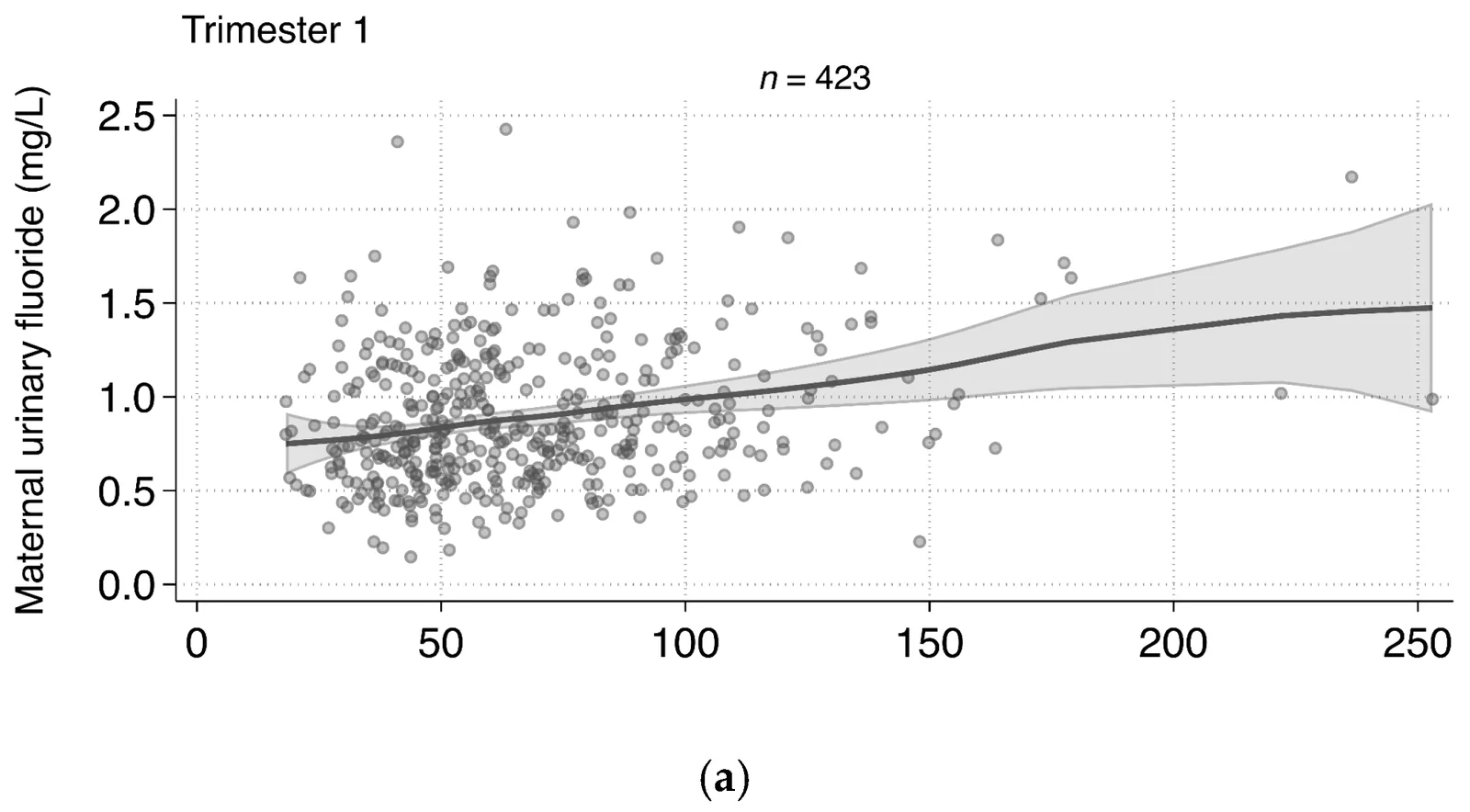

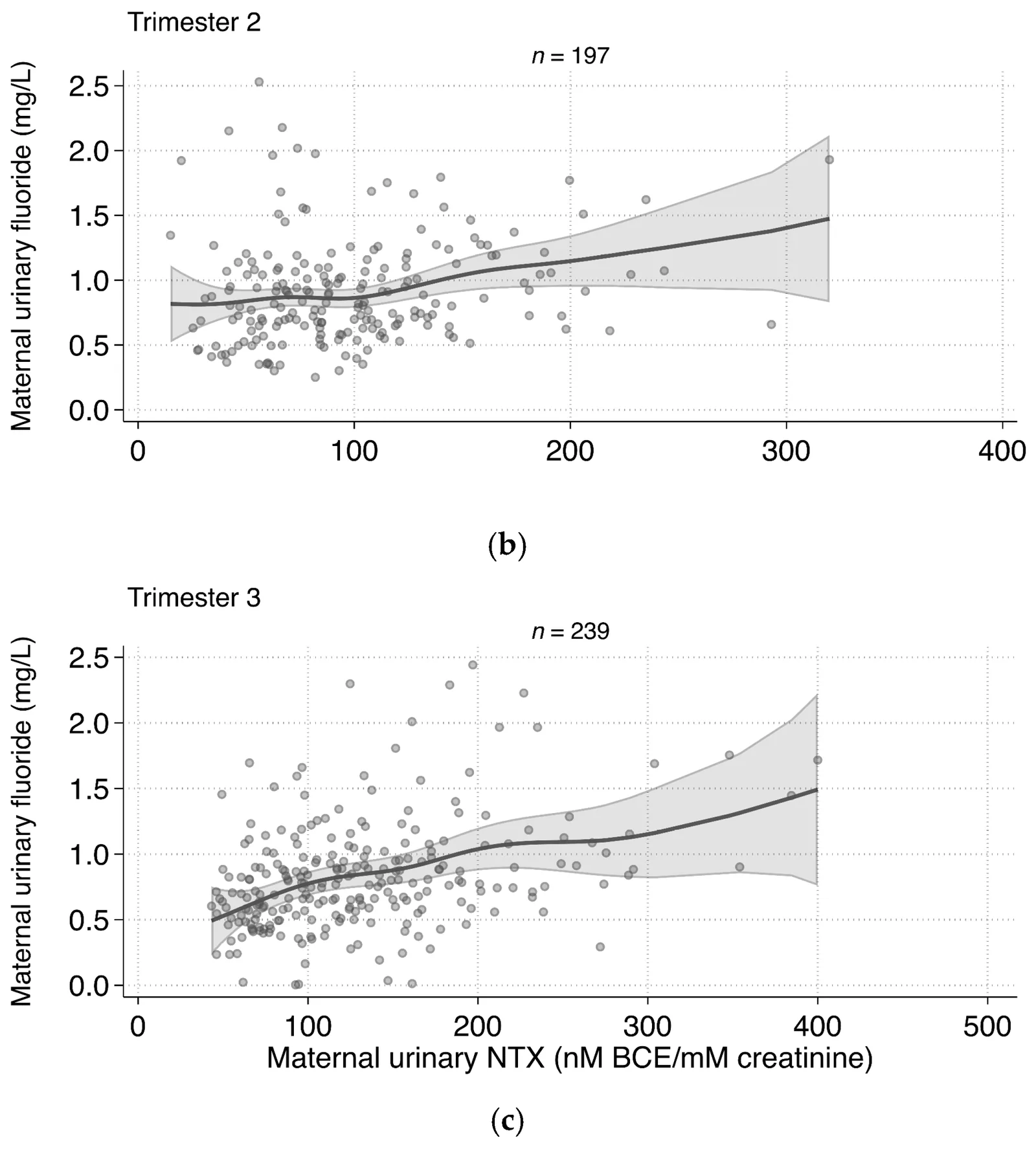
Generalized additive model (GAM) showing the association between maternal urinary N-telopeptide of type I collagen (NTX) and creatinine-adjusted maternal urinary fluoride (MUF) across pregnancy: (**a**) Trimester 1 (*n* = 423); (**b**) Trimester 2 (*n* = 197); (**c**) Trimester 3 (*n* = 239). Curves represent the adjusted relationship between NTX (nmol BCE/mM creatinine) and MUF (mg/L) for each trimester. Models are adjusted for maternal age, infant birthweight, firstborn status, and cohort membership (Cohort 2; Cohort 3 Ca–; Cohort 3 Ca+). Shaded areas represent the 95% confidence interval of the fitted smooth. Each point corresponds to an individual observation. Trimester-specific sample sizes are *n* = 423 (T1), *n* = 197 (T2), and *n* = 239 (T3). The x-axis is maternal urinary NTX (nmol BCE/mM creatinine); the y-axis is maternal urinary fluoride (mg/L).

Generalized additive models showed approximately linear relationships between NTX and MUF in all three trimesters, with no evidence of strong non-linearity. The quadratic NTX term was non-significant (*p* = 0.83, 0.38, 0.29), Figure 1.

In sensitivity analyses that additionally adjusted for estimated fluoride intake from water, beverages, and dietary sources, the magnitude and direction of the NTX–MUF associations were similar to those in the primary models, and all key inferences were unchanged. Excluding observations with high influence based on Cook’s distance did not materially alter effect estimates or *p*-values. GAM-based visualizations and formal tests for non-linearity led to the same conclusions as the primary models. After excluding the calcium-supplemented cohort, the NTX coefficients were 0.00300, 0.00167, and 0.00268 for trimesters 1–3 (*p* = 0.0001, 0.0499, and <0.001), with a wider 95% confidence interval for the attenuated second-trimester estimate in the smaller subsample (*n* = 149, after excluding 48 participants) (Table S2). Removing birthweight left estimates essentially unchanged (β = 0.00301, 0.00199, 0.00266; all *p* ≤ 0.001), as did replacing it with early-pregnancy maternal BMI (β = 0.00302, 0.00215, 0.00251; all *p* ≤ 0.001; Table S3).

## 4. Discussion

Maternal bone resorption, indexed by NTX, was significantly and independently associated with maternal urinary fluoride concentrations during pregnancy. Across all trimesters, NTX exhibited a positive and approximately linear association with MUF, and these associations persisted after adjustment for demographic factors, cohort membership, and estimated fluoride intake. The magnitude and consistency of these associations support a physiologically plausible mechanism in which skeletal fluoride, accumulated over years of environmental exposure, is mobilized during pregnancy-related increases in bone turnover [10]. These findings are consistent with endogenous fluoride released from bone contributing to urinary fluoride excretion [10,12]. This aligns with established mineral metabolism in pregnancy, where increased fetal calcium demand drives maternal skeletal resorption [17,18]. Given the substantial fraction of total body fluoride sequestered in bone [30], even modest elevations in resorptive activity can generate biologically relevant increases in circulating fluoride.

MUF may reflect integrated exposures over both short and longer time windows, and thus may capture cumulative fluoride dose rather than solely recent intake [10]. This finding is relevant for studies relating prenatal fluoride exposure to child neurodevelopmental outcomes [1,2,3,31], where more temporally stable exposure indices may strengthen causal inference.

Our study has a number of strengths, including the rigorous methods used to measure urinary fluoride and the use of a biological marker specific for bone resorption (NTX), and a large sample size of pregnant women who contributed data and samples at each stage of pregnancy. A limitation is that not all women had measures of MUF and NTX available at every trimester. Sample size was governed by creatinine-adjusted MUF availability rather than NTX; included and excluded participants did not differ demographically (Table S1). The cohorts differed in calcium supplementation, which lowers bone resorption [22]; the association was robust to excluding the calcium-supplemented cohort in the first and third trimesters and attenuated in the second, where the smaller sample reduced precision (Table S2), so residual confounding cannot be excluded. Estimates were unchanged after removing birthweight or replacing it with early-pregnancy maternal BMI, a temporally antecedent covariate (Table S3). We also had no measures of skeletal fluoride content, which could have allowed us to determine the extent to which such measures interact with bone resorption to influence MUF and fetal fluoride exposure. No instrument for making such measurements in vivo for epidemiological research is yet available. Finally, the design is observational and cross-sectional within trimester; the associations do not establish causation or quantify the bone-derived fraction of maternal or fetal fluoride.

Going forward, the insights from this study suggest that as the evidence implicating prenatal fluoride exposure as a neurodevelopmental toxicant grows, attention should be paid to maternal bone stores as a potential internal source of prenatal fluoride exposure. In addition to confirming our findings, future research is needed to assess the extent of the problem given that population exposure to fluoridated drinking water is widespread [32], as well as potential approaches for mitigating the likely neurodevelopmental impacts. We note that randomized placebo-controlled trials have shown that dietary supplementation during pregnancy with calcium decreases bone resorption [22,26] as well as blood lead levels [33]. Calcium supplementation has also been shown to decrease the risk of pre-eclampsia and is recommended by the World Health Organization for the many women (particularly in low- and middle-income countries) who have diets low in calcium [34,35]. However, the recommended doses are substantial (1.5–2 g/day), and a survey of public antenatal clinics in Brazil found that calcium supplementation was prescribed to fewer than 6% of women [36].

## 5. Conclusions

Maternal urinary fluoride during pregnancy appears to be influenced by endogenous skeletal fluoride release during bone resorption. MUF may therefore reflect both intake-derived and bone-derived fluoride, and its interpretation as an exposure biomarker should incorporate physiological context. Future epidemiologic studies should consider integrating bone turnover markers to improve exposure characterization and strengthen inference regarding fluoride’s developmental neurotoxicity. Attention is also needed to determine the extent to which bone stores of fluoride may pose an internal source of prenatal exposure to fluoride—and potential strategies for blunting such exposures and their impacts.

## Figures and Tables

**Table 1:** Descriptive characteristics of participants included in each trimester-specific MUF–NTX model, ELEMENT cohorts, Mexico City.

Trimester	*n*	MUF (mg/L) Mean (SD)	NTX (nmol BCE/mM cr) Mean (SD)	Age (yr) Mean (SD)	Birthweight (kg) Mean (SD)	Firstborn *n* (%)	Cohort 2 *n* (%)	Cohort 3 Ca– *n* (%)	Cohort 3 Ca+ *n* (%)
T1	423	0.89 (0.37)	68.0 (33.5)	26.5 (5.6)	3.14 (0.44)	148 (35.0%)	93 (22.0%)	171 (40.4%)	159 (37.6%)
T2	197	0.91 (0.41)	99.0 (50.3)	26.5 (5.5)	3.09 (0.45)	75 (38.1%)	102 (51.8%)	47 (23.9%)	48 (24.4%)
T3	239	0.83 (0.42)	132.4 (65.4)	26.6 (5.4)	3.13 (0.40)	87 (36.4%)	44 (18.4%)	106 (44.4%)	89 (37.2%)
All ^a^	506	0.89 (0.38) ^b^	69.9 (35.8)	26.6 (5.6)	3.13 (0.44)	178 (35.2%)	146 (28.9%)	185 (36.6%)	175 (34.6%)

MUF, maternal urinary fluoride (creatinine-adjusted, mg/L). NTX, N-telopeptide of type I collagen, reported as nmol BCE per millimolar of creatinine. Continuous variables: mean (SD); categorical variables: n (%). ^a^ All corresponds to the participant-level analytic sample (unique individuals with ≥1 trimester observation satisfying trimming and covariate completeness). ^b^ In the All row, MUF and NTX correspond to each participant’s earliest available trimester measurement. Cohort 3 Ca– is the placebo arm of the calcium randomized trial (reference cohort); Cohort 3 Ca+ is the calcium-supplemented arm; Cohort 2 is the observational pregnancy cohort.

**Table 2:** Trimester-specific association between maternal urinary NTX and creatinine-adjusted maternal urinary fluoride (MUF); Gamma generalized linear models with log link and robust standard errors, ELEMENT cohorts.

Trimester	*n*	β (NTX)	*p*-Value	1 SD NTX (nmol BCE/mM cr)	MUF Ratio per 1 SD NTX	% Increase MUF per 1 SD NTX	Absolute Δ MUF (mg/L)
T1	423	0.00315 (95% CI 0.00199–0.00431; SE 0.00059)	<0.0001	33.54	1.11 (95% CI 1.07–1.16)	+11% (95% CI 6.9–15.6)	+0.10
T2	197	0.00207 (95% CI 0.00088–0.00325; SE 0.00061)	0.001	50.32	1.11 (95% CI 1.05–1.18)	+11% (95% CI 4.5–17.8)	+0.10
T3	239	0.00253 (95% CI 0.00166–0.00341; SE 0.00045)	<0.0001	65.41	1.18 (95% CI 1.12–1.25)	+18% (95% CI 11.5–25.0)	+0.15

Trimester-specific Gamma GLMs (log link, robust SE). Outcome: creatinine-adjusted MUF (mg/L). Exposure: NTX (nmol BCE/mM creatinine). Covariates: maternal age, infant birthweight, firstborn status, and cohort (Cohort 3 Ca– reference). β reflects the log change in expected MUF per 1-unit increase in NTX; the 1-SD MUF ratio = exp(β × SD_NTX); % increase = (ratio − 1) × 100; absolute Δ MUF (mg/L) = mean(MUF) × (ratio − 1).

## Data Availability

Data availability is described in ELEMENT’s cohort description paper [25].

## Supplementary Materials

The following supporting information can be downloaded at https://www.mdpi.com/article/10.3390/toxics14090798/s1. Table S1: Baseline characteristics of participants included in versus excluded from each trimester-specific MUF–NTX model; Table S2: Calcium sensitivity: primary model versus model excluding the calcium-supplemented cohort (Cohort 3 Ca+), with 95% CI; Table S3: Covariate sensitivity for infant birthweight (a delivery-ascertained, post-exposure covariate): primary model, model with birthweight removed, and model with birthweight replaced by early-pregnancy maternal BMI; 95% CI.
