# Supplementary material for: Association of Maternal Bone Resorption with Urinary Fluoride Levels During Pregnancy
Source: Toxics. 2026 Sep 9;14(9):798. doi: 10.3390/toxics14090798 (PMC13611271; doi:10.3390/toxics14090798)
Supplement: Supplementary file 1 [file toxics-14-00798-s001.zip › toxics-4416313-supplementary.pdf]

Supplementary Materials

Association of Maternal Bone Resorption with Urinary Fluoride Levels During Pregnancy

Table S1. Baseline characteristics of participants included in versus excluded from each trimester-specific MUF–NTX model.

Characteristic	Included, mean (SD)	Excluded, mean (SD)	p
Trimester 1			
n (participants)	423	276	—
Maternal age, y	26.48 (5.62)	26.66 (5.36)	0.680
Birthweight, kg	3.14 (0.44)	3.07 (0.43)	0.065
Firstborn, %	35%	37%	0.653
NTX, nmol BCE/mM cr	68.0 (33.5)	81.6 (71.2)	0.003
Trimester 2			
n (participants)	197	511	—
Maternal age, y	26.46 (5.53)	26.82 (5.52)	0.445
Birthweight, kg	3.09 (0.45)	3.13 (0.41)	0.259
Firstborn, %	38%	35%	0.504
NTX, nmol BCE/mM cr	99.0 (50.3)	112.8 (93.7)	0.012
Trimester 3			
n (participants)	239	447	—
Maternal age, y	26.63 (5.43)	26.59 (5.51)	0.929
Birthweight, kg	3.13 (0.40)	3.15 (0.41)	0.673
Firstborn, %	36%	36%	0.987
NTX, nmol BCE/mM cr	132.4 (65.4)	158.1 (118.0)	<0.001

MUFcr, creatinine-adjusted maternal urinary fluoride. Excluded = NTX measured that trimester but not in the analytic model.

Table S2. Calcium sensitivity: primary model versus model excluding the calcium-supplemented cohort (Cohort 3 Ca+), with 95% CI.

Trimester	Primary β (95% CI)	Ca+ excluded β (95% CI)	n	p (Ca+ excl.)
T1	0.00315 (0.00199–0.00431)	0.00300 (0.00151–0.00449)	264	0.0001
T2	0.00207 (0.00088–0.00325)	0.00167 (0.00000–0.00334)	149	0.050
T3	0.00253 (0.00166–0.00341)	0.00268 (0.00164–0.00372)	150	<0.001

GLM (log link, robust SE); covariates: maternal age, birthweight, firstborn, cohort. β_NTX per model; 95% CI; n per trimester. Ca+, calcium-supplemented cohort (Cohort 3 Ca+).

Table S3. Covariate sensitivity for infant birthweight (a delivery-ascertained, post-exposure covariate): primary model, model with birthweight removed, and model with birthweight replaced by early-pregnancy maternal BMI; 95% CI.

Trimester	Model	β (95% CI)	n	p
T1	Primary (birthweight)	0.00315 (0.00199–0.00431)	423	<0.001
	Birthweight removed	0.00301 (0.00186–0.00416)	442	<0.001
	Early-pregnancy BMI	0.00302 (0.00184–0.00420)	432	<0.001
T2	Primary (birthweight)	0.00207 (0.00088–0.00325)	197	0.001
	Birthweight removed	0.00199 (0.00082–0.00316)	203	0.001
	Early-pregnancy BMI	0.00215 (0.00104–0.00327)	177	<0.001
T3	Primary (birthweight)	0.00253 (0.00166–0.00341)	239	<0.001
	Birthweight removed	0.00266 (0.00178–0.00353)	249	<0.001
	Early-pregnancy BMI	0.00251 (0.00157–0.00345)	235	<0.001

GLM (log link, robust SE); covariates: maternal age, firstborn, cohort, plus the covariate shown. BMI, early-pregnancy maternal body mass index.
